# The complete chloroplast genome sequence of *Phalaenopsis wilsoniii* (Orchidaceae)

**DOI:** 10.1080/23802359.2021.1994889

**Published:** 2021-10-27

**Authors:** Keke Xia, Ding-Kun Liu, Jie-Yu Wang

**Affiliations:** a BGI-Shenzhen, Shenzhen, China; b Key Laboratory of National Forestry and Grassland Administration for Orchid Conservation and Utilization at Colleage of Landscape Architecture, Fujian Agriculture and Forestry University, Fuzhou, China; c Key Laboratory of Plant Resources Conservation and Sustainable Utilization, South China Botanical Garden, Chinese Academy of Sciences, Guangzhou, China; d Laboratory for Orchid Conservation and Utilization, The Orchid Conservation and Research Center of Shenzhen, The National Orchid Conservation Center of China, Shenzhen, China

**Keywords:** Orchidaceae, Parishianae, moth orchid, plastid genome

## Abstract

In the present study, we reported and characterized the complete chloroplast genome of a moth orchid, *Phalaenopsis wilsonii*, which is endemic to South China. Its plastid genome size is 145,373 bp, consisting of a large single copy (LSC) region (84,996 bp), a small single-copy region (10,668 bp), and two inverted repeats (IRs) regions (24,855 bp). A total of 122 plastid genes were annotated, comprising 76 protein-coding genes, 38 tRNA genes, and 8 rRNA genes. The phylogenetic tree further revealed that *P. wilsonii* showed a sister relationship with *P. lowii* within subgenus *Parishianae*.

Moth orchid (*Phalaenopsis* Blume) is widely used in gardening around the world, and also occupies a large proportion of orchid industry (Van Huylenbroeck 2018). Previously phylogenetic analyses revealed that *Phalaenopsis* can be divided into four subgenera, subgen. *Phalaenopsis*, *Parishianae*, *Hygrochilus* and *Ornithochilus* (Kocyan and Schuiteman 2014; Li et al. 2014, 2016). Although Subgen. *Parishianae* constitutes more than a half of the species richness in *Phalaenopsis*, only one complete chloroplast genome of this subgenus can be found in NCBI (Wang et al. 2019). *P. wilsonii* is an endemic species in China, and is also a typical deciduous *Phalaenopsis* belonging to subgen. *Parishianae*. Here, we provide a complete chloroplast genome of *P. wilsonii*, aiming to facilitate our understanding on *Phalaenopsis* as well as to expand orchid resources in wild.

The sampling individual of *Phalaenopsis wilsonii* is cultivated in National Orchid Conservation Center in Guangdong province of China (114°19’01′’E, 22°60’34′’N), and a voucher specimen (noccphal031n) was also deposited in the Herbarium of National Orchid Conservation Center, Shenzhen, China. We extracted the total DNA from the young leaf of the voucher specimen and conducted high throughput sequencing at Illumina HiSeq 2000 platform (Illumina, San Diego, CA). Firstly, we constructed a reference dataset with all publicly available *Phalaenopsis* plastid genomes, and then mapped the clean reads against the reference dataset to obtain the chloroplast reads for *P. wilsonii*. PLATANUS (Kajitani et al. 2014) was adopted for contig assembly and scaffolding, resulting in the final complete genome with the artificial modification. BLAST was further used to align the reads onto the genome again to determine the IR boundaries, and the annotation was performed using Geneious 2019.0.3 (Kearse et al. 2012). The resulting complete chloroplast genome of *P. wilsonii* was submitted to GenBank under the accession number of MW218959. The total length of *P. wilsonii* chloroplast genome is 145,373 bp, with the GC content being 36.9%. The chloroplast genome we identified here is slightly shorter than other published moth orchids (from 146,834 bp for *P. lowii* to 148,964 bp for *P. aphrodite* subsp. *formosana*). As with other orchids, the chloroplast genome of *P. wilsonii* consists of a large single copy (LSC) region (84,995 bp) and a small single-copy region (10,668 bp), which are segmented by two inverted repeat (IRs) regions (24,855 bp). Overall, 122 genes (containing repeat region gene) were annotated, including 76 protein-coding genes, 8 rRNAs, and 38 tRNAs. Similar to a former study (Chang et al. 2006), all *ndh* genes of *P. wilsonii* are nonfunctional, and the *ndhE* was also missing.

To infer the phylogenetic relationship for *P. wilsonii*, we used RAxML v.8 (Stamatakis 2014) to reconstruct the maximum-likelihood phylogenetic tree based on the whole plastid genomes of *P. wilsonii*, four moth orchids, and also the other ten orchids. *Cattleya crispate* was applied as an outgroup according to the topology from Givnish et al. (2015). The TIM1 + I + G model was applied by jModelTest (Posada 2008), and the reliability of topology was supported with 1000 bootstrap replicates. Consistent with Givnish’s study Givnish et al. (2015), the phylogenetic tree showed that Vandeae presented a sister relationship to Cymbidieae. Besides, *P. wilsonii* was sister to *P. lowii*, both of which belong to the subgen. *Parishianae* that are grouped into a single clade (Figure 1). This complete chloroplast genome of *P. wilsonii* will be helpful for future phylogenetic studies and conservation in *Phalaenopsis*.

**Figure 1.**
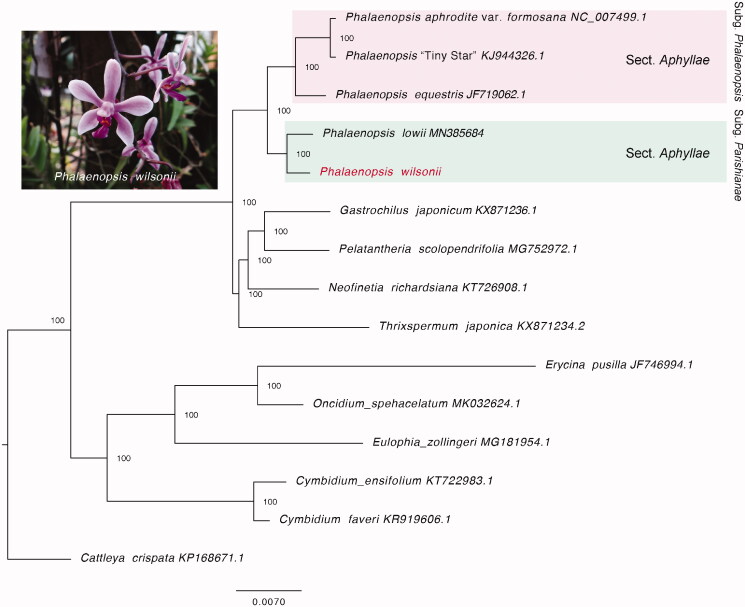
Maximum-likelihood tree reconstructed by RAxML based on complete chloroplast genome sequences from *P. wilsonii*, four *Phalaenopsis* species, and ten other orchids. *Cattleya crispate* was selected as an outgroup. Numbers on branches are bootstrap support values.

## Data Availability

The data that newly obtained at this study are available in the NCBI under accession number of MW218959 (https://www.ncbi.nlm.nih.gov/nuccore/MW218959). The sequencing reads are available under the SRA accession number of SAMN18924765.
